# Neuroendocrine System Adaptation during Consecutive Extrinsic Stimuli: A Pilot Dynamic Study

**DOI:** 10.3390/children10020248

**Published:** 2023-01-30

**Authors:** Styliani A. Geronikolou, Vasilis Vasdekis, Aimilia Mantzou, Constantinos Davos, Dennis V. Cokkinos, George P. Chrousos

**Affiliations:** 1 Clinical, Translational Research and Experimental Surgery Centre, Biomedical Research Foundation of the Academy of Athens, 4, Soranou Ephessiou Str., 11527 Athens, Greece; 2 First Department of Pediatrics, National and Kapodistrian University of Athens Medical School, “Aghia Sophia” Children’s Hospital, Thivon 1, 11527 Athens, Greece; 3 University Research Institute of Maternal and Child Health and Precision Medicine, National and Kapodistrian University of Athens Medical School, Levadias 8, 11527 Athens, Greece; 4 Department of Statistics, Athens University of Economics and Business, Kodrigtonos 12, 11257 Athens, Greece

**Keywords:** Trier Social Stress Test for Children, HPA axis, electromagnetic fields, cell phone call, low-level inflammation, non-linear dynamics, heart ANS, sample entropy

## Abstract

This pilot repeated measures study aims to evaluate the dynamics of the autonomic nervous system (ANS), the hypothalamic–pituitary–adrenal (HPA) axis, and/or their interplay with low-level inflammation in healthy schoolchildren during consecutive extrinsic stimuli. Twenty healthy schoolchildren and adolescents aged 11–14 years (12.5 ± 1.5) were consecutively exposed to an oral task (#2) and an arithmetic task (#3) (Trier Social Stress Test for Children (TSST-C)), lasting 5 min each, and a three-minute cellular phone call (#4). Salivary cortisol (SC) was sampled at baseline (#1) and immediately after each exposure (#2, 3, and 4). Baseline serum high-sensitivity C-reactive protein (hsCRP) and cortisol levels were also assessed. ANS dynamics and complexity were measured using Sample Entropy (SampEn) at each experimental time period (#1–4). Baseline serum hCRP and cortisol correlated negatively to each other, while the ANS and HPA axis acute reactions to the three consecutive stimuli differed over time. The ANS adaptation to these stimuli included complexity modulation, which was not dependent on baseline hsCRP or cortisol, and weakened during the third stimulation. However, baseline hsCRP and cortisol had a weakening and an increasing effect on the HPA axis over time, respectively. We conclude that low-level inflammation and baseline morning cortisol level have no effect on ANS dynamics but influence the HPA axis response to consecutive external stimuli.

## 1. Introduction

Human survival depends on homeostatic maintenance achieved only after incessant adaptations to extrinsic/intrinsic stimuli. Extrinsic stimuli include stressful natural, lifestyle, and psychosocial challenges. Psychosocial stress, as well as exposure to electromagnetic fields, are common environmental “hazards” that are progressively gaining research interest. Environmental challenges are faced by a complex adaptive body mechanism described as “fight, flight or freeze” response, involving the autonomic nervous system (ANS), the hypothalamic–pituitary–adrenal (HPA) axis, and the inflammatory reaction [1].

The HPA axis, which constitutes a key stress response system of the organism, may influence the activities of basically all organ systems, including the nervous, cardiovascular, and immune systems [2]. Salivary cortisol is a non-invasive and sensitive stress biomarker with a high correlation to serum cortisol levels [3,4]. This biomarker has been repeatedly used in the Trier Social Stress Test for Children (TSST-C) [5,6], which is an experimental tool for stress assessment irrespective of an individual’s confounding factors, such as personality type, education, socioeconomic status, etc. [7]. The TSST-C consists of public speaking and a mental arithmetic task in front of an audience (usually the investigators). On the other hand, the ANS is also activated during acute stress [8], with immediate secretion of the catecholamines norepinephrine and epinephrine followed by secretion of interleukin-6, an inflammatory cytokine [9,10]. The latter stimulates the circulating levels of high-sensitivity C-reactive protein (hsCRP) [11,12]. Notably, according to recent research, cardiovascular diseases (CVD) have their start and origin to some extent in the early developmental stages of life (intrauterine life, childhood, and puberty) [13,14]. Heart rate variability (HRV) is a valid marker of ANS activity, as it expresses the combined effects of the sympathetic and parasympathetic limbs of the ANS on the heart and is predictive of cardiac pathology. 

The ANS and the HPA axis have been extensively investigated before and after extrinsic stimulation. The HPA axis response is credibly detected by salivary cortisol levels twenty minutes after stimulation. Yet, the dynamics regarding a significant cortisol change (rise or decrease) during stress has been scarcely investigated. The synchronous study of the ANS and HPA have been investigated even less, while the relevant research is inter-associated with the vagal tone in the supine posture [15,16].

Importantly, the homeostatic crosstalk of ANS, the HPA axis, and inflammation in relation to repeated stress includes adaptation processes, whose dynamics are also little known and understood [17]; however, habituation after similar stressors or sensitization has been reported [17,18]. Notably, the dynamics taking place during consecutive and differing stressors have been investigated only in a rudimentary fashion, whilst only few population studies have established a correlation of pro-inflammatory markers (Tumor Necrosis Factor alpha, TNF-a, or interleukin 6 (IL-6) with cortisol release in adults [19]. However, to date, no study in children and adolescents on the correlation of hsCRP with salivary cortisol concentrations upon and/or after successive extrinsic challenges has been reported.

In a previous study, we investigated the HPA axis response 10 and 20 min after mental stress and/or a cell phone call [18]. However, the HPA axis in parallel with the ANS (HRV) dynamics and complexity during consecutive extrinsic stimuli have not been investigated yet. Furthermore, the effects of low-grade inflammation or baseline circulating cortisol in this situation have not been evaluated either. In this study, we aimed to cover the above stated gaps: to detect not only the HPA axis and ANS dynamics of the acute stress response during consecutive external stimuli, such as a combined mental task and a cellular phone call in childhood and early puberty, but also to examine the possible modulating influence of baseline low-grade inflammation or cortisol on these effects.

## 2. Materials and Methods

### 2.1. Subjects

Twenty healthy schoolchildren aged 11–14 years (12.5 ± 1.5) were enrolled in this pilot study. Inclusion criteria were normal body weight, absence of infection during the previous month, absence of any cardiac disease or other chronic medical condition, or taking a chronic medication. A careful clinical examination (including body weight, height, and body mass index, all expressed in z-score) was conducted by an experienced pediatrician and personal medical history data were collected. Venous blood sampling for hsCRP and cortisol assessment was performed after overnight fasting at 8.00 am. 

The study population was evaluated at the Cardiovascular Research Laboratory of the Biomedical Research Foundation of the Academy of Athens. The study protocol adhered to the Helsinki Guidelines of Good Clinical Practice and was approved by the Ethics Committee of the Aghia Sophia Children’s Hospital. A signed informed consent was obtained from the children’s parents/guardians before the children were enrolled in the study. 

### 2.2. Exposure

#### 2.2.1. Trier Social Stress Test for Children (TSST-C)

We followed the previously proposed Trier Social Stress Test for Children (TSST-C) procedure [18]: “Upon arrival in the laboratory, the participants rested for 20 min. At time +15 min, the researcher re-entered the room asking the participant to introduce himself or herself. The child was told that he/she should complete the following story as excitingly and plausibly as possible—better than all other children. “You decided to make a Sunday trip to a forest with your friends. The sun was shining and you climbed the mountain Taygetos (a mythical Greek mountain with dangerous itineraries and unexplored forests). In the afternoon, just before you decided to return, clouds appeared in the sky, and soon afterwards, a tremendous rainfall began. The pathways disappeared under the unexpected rainfall and you found yourself wet, lost from the rest of the group, without guiding pathways or signs, with no possibility to lodge or protect yourself. Darkness dropped around you and huge rolling rocks started falling from the top of the mountain. The blowing wind and thunders did not allow you to hear your colleagues’ voices; you are lost”. The investigator quits the room again and returns 2 min later to hear the narration of the child. This constituted the narrative part of the test. Afterwards, the investigator asked the participant to count down from a large prime number (1492) in decrements of 13 as quickly and accurately as possible. On every failure, the child had to restart at the beginning number with the investigator prompting:“error—restart”. Five minutes later, the task was completed and the investigator left the room.”

A calming period lasting 5 min followed the arithmetic task before the 3 min cellular phone call, in an attempt to simulate real life events; we assumed that the consecutive stressors were additive.

#### 2.2.2. Cellular Phone Call Exposure

The cellular phone call of 3 min duration was conducted after the 5 min relaxation period following the TSST-C. The participants rested in the supine position in the same laboratory setting and exposure circumstances as previously described [20]. The exposure sources were 2G and 3G phones, keeping the radiofrequency exposures identical, whereas parents were instructed to talk while making this call to their child. The Specific Absorption rate ranged from 0.268–1 W/kg (under the ICNIRP limits [21]). 

### 2.3. Measurements

#### 2.3.1. Blood Sampling

Blood samples were collected at baseline after 12 h of fasting (1–1.5 h after awakening).

#### 2.3.2. Autonomic Nervous System (ANS)

Autonomic nervous system recordings were performed by Task Force Monitor in supine position, in a comfortable, quiet, and metal-free laboratory room, during baseline period (#1) lasting 30 min, the oral task of TSST-C (#2) lasting 5 min, the arithmetic task of TSST-C (#3) lasting 5 min, and the mobile phone call (#4) lasting 3 min. Participants had been instructed not to have had breakfast (chocolate, tea, coffee that might confound sympathetic activity, etc.). 

#### 2.3.3. Saliva Sampling 

The saliva samples were collected at baseline and after the completion of each stressor: during baseline calm state before TSST-C initiation (sample #1), directly after the oral (sample #2) and the arithmetic (sample #3) task, and after a 3 min cellular phone call (sample #4).

### 2.4. Laboratory Assessments

#### 2.4.1. Blood Samples 

Within 20 min after venipuncture and blood collection in EDTA tubes, the samples were centrifuged in 4 °C at 2000× *g* for 20 min and divided into aliquots. The aliquots were then stored at −80 °C in the Biomedical Research Foundation of the Academy of Athens until analysis. Hormonal and biochemical measurements were performed in the Endocrinology Laboratory at the Choremeion Research Institute of the First Dpt. of Pediatrics of the National and Kapodistrian University of Athens Medical School. The serum cortisol values were assessed by an Immunochemiluminescence assay (Immulite 2000 Siemens). 

#### 2.4.2. Salivary Cortisol Samples

Salivette saliva collection devices (Stardsted, Norbrecht, Germany) were used for Salivary cortisol sample collection. Immediately after collection, they were centrifuged at 4 °C at 2400× *g* for 20 min, and aliquots were kept at −80 °C in the Biomedical Research Foundation of the Academy of Athens. The samples were sent to the Choremeion Endocrinology Laboratory of “Aghia Sophia” Children’s Hospital (Athens, Greece) on dry ice upon study completion. The salivary cortisol analysis was performed by a Chemiluminescence assay in a Roche COBAS E411 analyzer.

### 2.5. Data Analysis 

The sample size was chosen according to the rules of thump suggested by Julius [22] and/or Keiser and Wassmer [23], included in the reference by Whitehead et al. [24]. Results are presented as mean ± standard deviation. Circulating and saliva concentrations were analyzed with the Shapiro–Wilk test to test the normality of their distribution. ANOVA for repeated measurements (RANOVA) was used to estimate the fluctuation of the four salivary cortisol levels. Analysis of covariance for repeated measures (ANCOVA) was also applied to find possible predictors of cortisol response at each time point of the study. Power of the analysis of covariance was conducted with SPSS multivariate tests (ANCOVA). Multiple covariate effects on salivary cortisol concentrations between subgroups were also assessed. Contrast evaluations with repeated contrasts were performed to estimate alterations between each time point of the procedure. The Greenhouse–Geisser correction was applied to account for the violation of the sphericity assumption. 

The Pearson correlation coefficient was calculated to confirm all biochemical correlations. A mixed effects analysis was conducted using an AR(1) covariance model for the errors along with time effects, covariate effects, and time by covariate interaction effects. As covariates, the baseline circulating hsCRP and cortisol measurements were used. 

Non-linear computations of ANS (heart rate variability (HRV)) dynamics and complexity were performed with MATLAB software. SampEn was introduced as a measure of complexity and regularity of brief time series that corrects Approximate Entropy [25] inherited bias, and thus becomes sensible and credible for very short and noisy time series (<200 beats). SampEn (m, r, and N) is defined as the negative natural logarithm of the conditional probability that two sequences within a time series of length *N* that are similar for *m* points remain similar for *m* + 1 points, within a tolerance of *r* [26]. We opted to use *m* = 2 and *r* = 0.2, as proposed in literature for HRV analysis [27]. The computed SampEn for each time was statistically analyzed with RANOVA, same as endocrine measurements. Its mean value covariance with baseline circulating hsCRP and cortisol were evaluated with RANCOVA, while effect sizes were calculated as well. The statistical significance *p* value was set at <0.05. Statistical analyses were performed by SPSS Version 17 Software.

## 3. Results

The participants characteristics assessed at baseline are described in Table 1. 

### 3.1. ANS Dynamics

**Figure 1:**
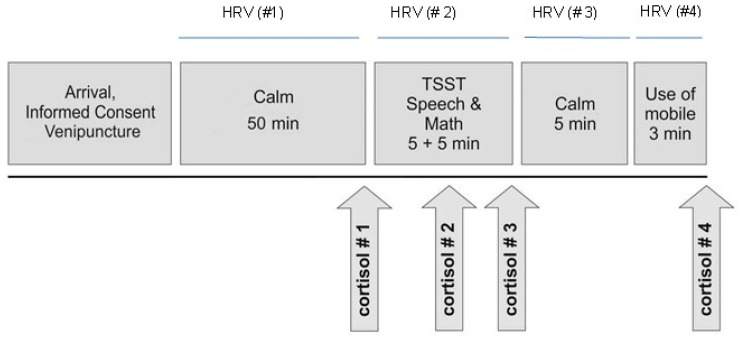
Study design (repeated measures of salivary cortisol and heart rate variability.

**Figure 2:**
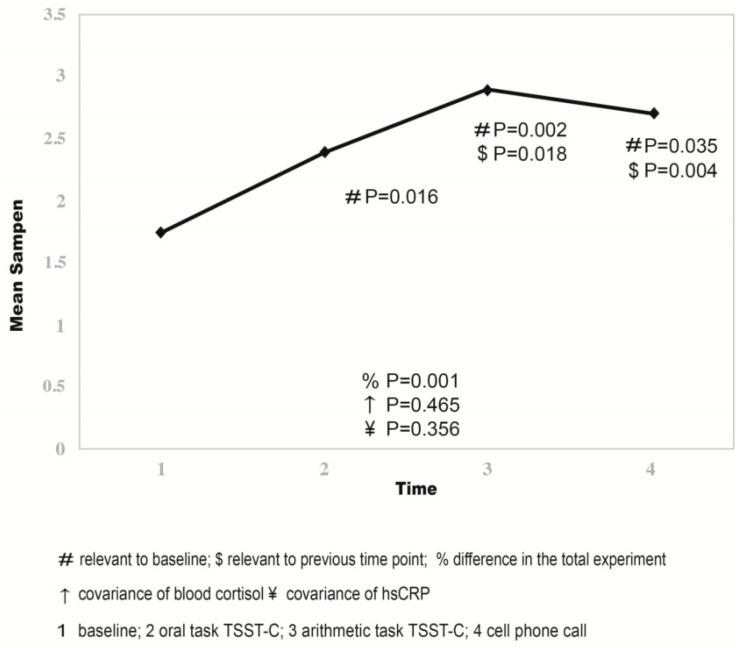
Mean SampEn in each experimental period.

The time phases (#1–4) when heart rate variability (HRV) was recorded are described in Figure 1. The mean computed SampEn observed during the investigational phases is presented in Table 2 and Figure 2. SampEn changed significantly (F = 217,82, *p* < 0.001, and η^2^ = 0.948) with an observed power of 1.00. The 95% CIs for each time period were (#1) [1.64, 1.85], (#2) [1.9, 2.1], (#3) [2.15, 2.33], and (#4) [2.06, 2.24] (Figure 2). A non-significant effect of covariance of baseline hsCRP or cortisol to SampEn was calculated, and the following were also calculated: F = 0.924, *p* = 0.356, partial eta squared 0.071, and observed power 0.144 for the hsCRP effect; F = 0.469, *p* = 0.465, partial eta squared 0.045, and observed power 0.107 for the cortisol effect.

### 3.2. HPA Response Dynamic

**Figure 3:**
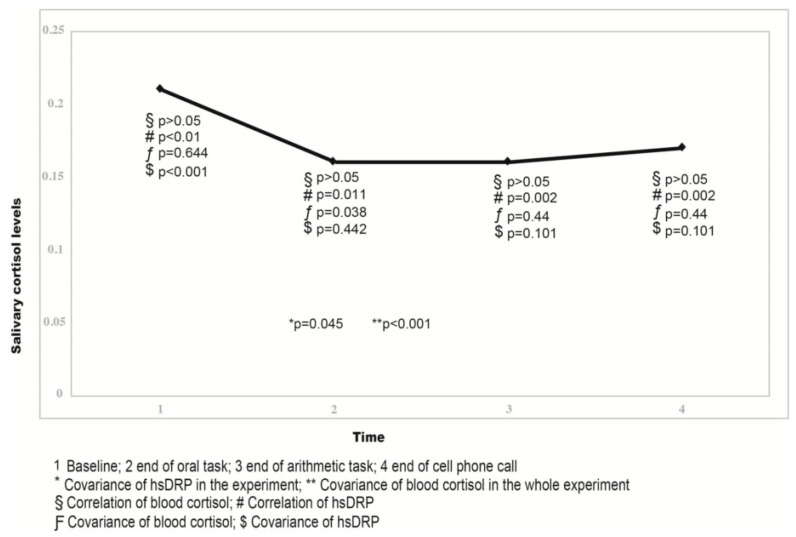
Salivary cortisol levels (μg/dL) during exposure and its covariance with baseline circulating hsCRP and cortisol.

The salivary cortisol levels on each investigated phase are presented in Table 3 and illustrated in Figure 3. 

The Shapiro–Wilk test for normality confirmed the salivary cortisol distribution’s normality (*p* > 0.05). Mauchly’s test (W = 0.091) indicated that the assumption of sphericity had been violated (x^2^(5) = 31.232, *p* < 0.001), therefore degrees of freedom were corrected using the Greenhouse–Geisser estimates of sphericity (ε = 0.487).

The salivary cortisol levels did not change significantly from baseline throughout the different test stages. However, a mild decrease in salivary cortisol levels was noted during mental stress exposure, and this reached limited statistical significance (F(3) = 4.329, *p* = 0.058, η^2^ = 0.72). Salivary cortisol response to mental stress is expected to be visible 10–20 min after stress is initiated. Thus, a slight though non-significant decrease in salivary cortisol during the 3-min cellular phone call was observed (Figure 3). Salivary cortisol concentrations at all time points were not correlated with anthropometric parameters, such as height, body weight, BMI, BMI z-score, gender, or age. The results of the repeated analysis of covariance (RANCOVA) are illustrated in Figure 2.

Estimates of covariance matrix parameters are shown in Table 4 and Table 5. The AR(1) mixed model confirmed the RANCOVA and Pearson coefficient in that there was a statistically significant interaction between time and baseline circulating cortisol (F(1) = 3.275, *p* = 0.033). Therefore, the effect of baseline circulating cortisol is practically the same as that at baseline (time 1) and became stronger as stress evolved (subsequent time points) (Table 4).

The salivary cortisol concentration during the call was inversely correlated to the baseline circulating cortisol in this sample (r(15) = −0.656, *p* = 0.008) (Figure 3).

**Figure 4:**
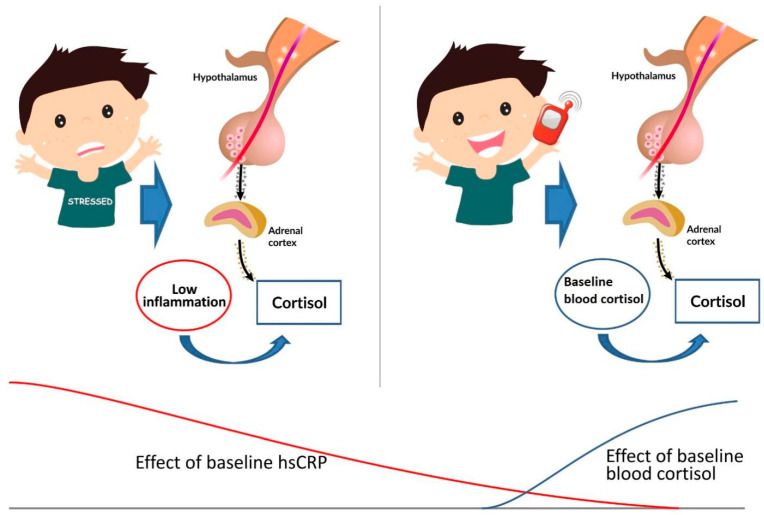
The effect of baseline circulating hsCRP and cortisol on salivary cortisol dynamics during consecutive stimuli.

A significant effect of covariance of baseline circulating hsCRP to salivary cortisol levels during all time points was observed (F(1) = 17.785, *p* < 0.001, η^2^ = 0.39, observed power 0.996) (Figure 3). The Pearson correlation of baseline circulating hsCRP to salivary cortisol at baseline was r(15) = 0.854, *p* <0.001, g_1_=−3.116, to salivary cortisol immediately after speech r(15) = 0.636, *p* = 0.011, and to salivary cortisol level immediately after math r(15) = 0.732, *p* = 0.002 (Figure 3). A limited effect of covariance of baseline circulating hsCRP on salivary cortisol levels during the call was observed (F(1) = 5.425, *p* = 0.045 η^2^ = 0.146, observed power 0.685) (Figure 3 and Figure 4).

Due to the result derived by the RANCOVA, we created a new mixed model AR(1) that confirmed the statistically significant interaction between time and hsCRP (F(1) = 13.752, *p* < 0.001) (Table 3). We further estimated the power of each effect at each time point and concluded that the effect of baseline circulating hsCRP is strong at time 1 and becomes weaker as time progresses (Table 3). The differences in slopes with the time points are shown in Table 4 (for baseline circulating hsCRP covariate) and Table 5 (for baseline circulating cortisol covariate). The estimated linear effect of baseline circulating hsCRP at the final time point (time = 4) was weaker: −0.0093 (SE = 0.0048, *p* = 0.065) (Table 5). These results were confirmed by the analysis of covariance. After confirming the baseline circulating hsCRP level covariance, we further tried to detect a cut-off point beyond which the effect of low inflammation covariance vanishes. A 75% quartile of more than 1.663 SIU mg/L of hsCRP levels was detected, with limited significance (*p* = 0.057). 

## 4. Discussion

The repeated measures design was opted for because it allowed statistical credibility with relatively few subjects [28]. This kind of design is generally feasible for pilot studies like this. Our population was healthy, lean, and homogeneous with regards to age range and anthropometry. The samples were taken at the completion of each stimulation. In addition, the subjects enrolled in this study were different from those that participated in an earlier similar study [18]. Moreover, we employed the previously translated into Greek and adapted to Greek children culture/experience TSST-C version, which we validated and used in our earlier study [18]. TSST has been previously used in a publication studying both the HPA axis and ANS in adults [29].

### 4.1. Dynamic of the ANS Response

The cardiac ANS measure of the HRV is a valid marker of a physical and emotional state [30], comprising stochastic interactions of complex physiological processes; thus, linear methods seem to be a drawback and non-linear methods such as Entropy parameters (i.e., Approximate Entropy, ApEn, Sample Entropy, SampEn) are more sensitive tools for its evaluation [31]. Moreover, the non-linear analysis option was imposed by the design of this study, as well as the experimental circumstances: such an analysis is feasible for dynamic and complex investigations, excluding both physiological noise due to stochasticity and EMF influence on monitoring devices. We evaluated Sample Entropy (SampEn) that corrects Approximate Entropy (ApEn) bias [32,33], becoming credible for even short recordings (<200 beats) [34,35]. It is insensitive to changes in trends in R-R intervals, unravelling encrypted patterns even in short-term and ultra-short-term recordings, like ours, and provides the required flexibility to study uncertain and changing adaptive situations [36]. 

Our study showed the following: (a) increased SampEn in all exposure phases (relevant to the baseline resting period before extrinsic stimulations), thus increasing uncertainty and complexity over time (#2,3,4) (*p* = 0.0014) and decreased predictability relevant to baseline; (b) a modestly decreased SampEn during the third exposure relevant to the previous one. In other words, our study showed that our subjects’ heart function increases in complexity during consecutive environmental stress challenges, perhaps in an effort to adapt and counterbalance allostatic load. A recent study by Mestanikova [37] had similar results regarding normal schoolchildren, where participants showed increased complexity in HRV dynamics during stress [37].

Unlike the Ahamed et al. results [27], our experiment showed slightly increased disorder (SampEn) during the cell phone call relevant to the baseline resting period. The explanation may lie on the experimental design employed: the cell phone call exposure came after mental stress and a very short resting period, which was not enough for the ANS to recover from the previous stimuli. The call acted as a supplementary stimulation, though children exhibited variability in irregularity during this last period, meriting further investigation with different metrics. Our findings are consistent with those by Yilmaz and Yildiz [38] where another non-linear parameter, the Largest Lyapunov Exponent, was opted for in evaluating a different population type (young adults). In addition, our participants showed a modest decrease in irregularity during the third stimulation (phone call) relevant to the previous mental stress: It is established that SampEn is positively associated to the vagal tone in the supine position [15,16]. Our study revealed an increase in the vagal tone during stress in the supine position, which was previously observed and established in adults [39] and children [40]: this has been associated with greater anxiety and less perceived control over anxiety. Thus, we hereby observe that HRV habituates after consecutive stimuli. 

High resting cardiac vagal control (like the one children exhibit in the supine position) has been associated with a sympathetic tone enhancement (imposed by increased unpredictability) as a response to stressors and is suggested to represent a flexible (highly adaptive) system [37,41], consistent with the early age (childhood/puberty) and developmental stage of our participants.

### 4.2. Dynamic of the HPA Response

In this study, we demonstrated that the salivary cortisol concentrations at baseline and during three consecutive stressors showed minor differences, indicating that each participant may have had a different reaction to each of the consecutive stimuli. These results may be attributed to the marked inter- and intra-individual variability and the adaptivity of the HPA axis. The observed decreased salivary cortisol levels during mental stress have previously been related to downregulated sensitivity of the endocrine system [42], externalizing behavior [43], bullying, aggressiveness, insecure attachment [5], parental divorce, and/or lower income [44]. As mentioned in a previous work of ours, this latter contingency may be related to the current socioeconomic crisis that influences our children’s families in Greece.

Our population consisted of volunteers who were recruited in a brief period, with an equal male/female ratio. Kudielka et al. in 2004 [45] indicated that there was no impact either of gender or of age (adults vs. children) on the salivary cortisol response. The evaluation of baseline circulating cortisol concentration as a predictor of the HPA axis response to stress (even if the cellular phone is assumed as an additive environmental triggering factor) reveals the influence of chronic stress. The attenuated response to acute mental stress may indicate HPA axis desensitization [46,47]. 

We also demonstrated that the acute reaction of the HPA axis, such as during mental stress and a cellular phone call, was related to baseline circulating hsCRP. It has been previously shown that repeated exposure even to mild stressors is sufficient to inhibit the effect of inflammation in animal models [48], suggesting a link between stress stimulation and intracellular inflammatory signaling. A recent young male population study reported increased G-protein-coupled receptor kinase-2 (GRK2) expression in peripheral blood mononuclear cells (PBMC) following an acute stress task [49].

The effect of circulating hsCRP decreased over time, contrary to the baseline circulating cortisol effect that increased (Table 4 and Table 5, Figure 4); thus, the HPA axis reaction to the third stimulation—the 3-min cellular phone call—depended on the baseline circulating cortisol levels [50,51]. It is highly associated with entities such as obesity [52], renal failure, airway inflammation [53], and autoimmune diseases [54], which were a priori excluded in our study. As far as children and schoolchildren are concerned, hsCRP concentration has also been reported to be associated with sleep-disordered breathing. The additional threshold detected (if hsCRP levels were higher than 1.663 mg/L, the effect of covariance tended to become apparent over time) should be checked in larger-sized samples. This would probably provide us with a narrower percentile or accurate cut-off point. Notably, the baseline circulating markers hsCRP and cortisol negatively correlated with each other, with a correlation coefficient of −0.632 and a significance level *p* = 0.021, confirming the contradictory dynamics observed during consecutive extrinsic stimuli, as shown herein.

Our study has to be considered in the light of its limitations. The participants were healthy, had a normal body weight, and were aged 11–14 years. Great effort was invested in designing an experimental protocol that would simulate stressors in the most realistic way. We focused on the acute response to a cellular phone call after mental stress stimulation. The power of the analysis of covariance was the greatest for the hsCRP and acceptable for the circulating cortisol. However, the power of our investigation’s results makes our study feasible and suggests that a larger sample of children matched with adult populations, where subgroup comparisons would be allowed, is feasible (i.e., larger populations, children vs. adults, etc.). Furthermore, a longer follow-up study with end-point data is necessary to determine if hsCRP and circulating cortisol levels can be used as predictors of future cardiovascular disease. On the other hand, in our study we attempted to reveal the dynamics of the response during consecutive stimuli as in real life, rather than the cortisol peak. 

To our knowledge, a prospective evaluation of the influence of a low-grade inflammation marker (hsCRP baseline circulating levels) on salivary cortisol (HPA axis biomarker) response during mental stress, as well as during a cellular phone call, has not been reported. Therefore, the pathophysiologic link between acute HPA axis reaction, CRP gene variation, and cellular phone use or mental stressors merits further evaluation in the future.

## 5. Conclusions

This effect of baseline circulating low inflammation (hsCRP) on salivary cortisol levels decreases over time, contrary to the baseline circulating cortisol effect, which increases. Furthermore, the respective cut-offs set as risk factors for future cardiovascular disease development in adults seem not to apply in our sample group. This could be attributed to the young age and/or the significant effect of covariates. The detected cut-off point elucidates a trend worth evaluating in a future study including a larger population. 

In addition, we showed that participants exhibited clear increasing complexity in cardiac function during the double tasks of TSST-C and a slightly more modest increase in the third stimulation (cell phone call) as well. The ANS (HRV) dynamics differed significantly during all stages of this study, illustrating its flexibility and adaptivity. Moreover, a loss of perceived control of stress due to anxiety provoked by the mental task was shown. Setting off an extrinsic stressor as expressed by the ANS dynamics expressed by SampEn was not modulated by baseline circulating markers (hsCRP or cortisol) in this study.

In other words, this study illustrates the dynamics and plasticity of a body response during consecutive common extrinsic challenges, potentially serving as a reference pilot study for future environmental health and/or pediatric investigators.

## Figures and Tables

**Table 1:** Participant characteristics assessed at baseline (mean ± SD).

Age (years)	12.5 ± 1.5
Μales	11 (52%)
Females	9 (48%)
BMI	19.1 ± 3.11
BMI z-score	0.23 ± 1.27
Height	154.57 ± 10.73
Weight	46.13 ± 8.27
Blood cortisol (μg/dL)	12.84 ± 5.96
hsCRP SIU (mg/L)	1.77 ± 0.703
SAR (W/kg)	0.268–1.00

**Table 2:** Mean Sample Entropy during baseline and consecutive exposures ± SD (n = 18).

	50 min Baseline	5 min Oral Task (during Speech)	5 min Arithmetic Task (during Math)	3 min Cell Phone Call
SampEn	1.74 ± 0.1	2.22 ± 0.3	2.75 ± 0.1	2.56 ± 0.2

min: minutes, SampEn: Sample Entropy, SD: standard deviation.

**Table 3:** Salivary cortisol levels (μg/dL) during exposure in the whole population (mean±SD, Confidence Intervals, CI, 95%) (n = 20).

	Baseline	0 min after Oral Task (during Speech)	0 min after Arithmetic Task (during Math)	0 min after Phone Call (during Call)
	0.21 ± 0.177	0.16 ± 0.09	0.157 ± 0.05	0.167 ± 0.09
	95% CI	95% CI	95% CI	95% CI
	[0.102, 0.325]	[0.108, 0.223]	[0.13, 0.193]	[0.108, 0.223]

**Table 4:** Differences in slopes of the first time point sampling with the three following points (covariance parameter baseline circulating (blood) hsCRP/cortisol) (n = 20).

hsCRP		Difference of Slopes of Each Time with Time 1	s.e	*p*-Value
	Time 2	−0.1219	0.0214	<0.001
	Time 3	−0.1522	0.0280	<0.001
	Time 4	−0.1724	0.0318	<0.001
**Baseline Circulating Cortisol**		**Difference of Slopes of each Time with Time 1**	**s.e**	***p*-Value**
	Time 2	0.0067	0.0031	0.040
	Time 3	0.0103	0.0044	0.023
	Time 4	0.0025	0.0049	0.611

**Table 5:** Estimates of covariance parameters baseline circulating hsCRP and cortisol (n = 20).

Parameter Circulating hsCRP		Estimate	Std. Error	Wald Z	Sig	95% Confidence Interval
Repeated Measures	AR1 diagonal	0.00591	0.00168	3.513	0.000	[0.00338, 0.01033]
	AR1 rho	0.70571	0.10166	6.941	0.000	[0.44754, 0.855293]
**Parameter circulating cortisol**		**Estimate**	**Std. Error**	**Wald Z**	**Sig**	**95% Confidence Interval**
Repeated Measures	AR1 diagonal	0.01225	0.00394	3.107	0.002	[0.006522, 0.02302]
	AR1 rho	0.78398	0.08421	9.310	0.000	[0.556207,0.90220]

## Data Availability

The data are restricted by GPDR, and written informed consent was obtained.
